# One-Step Formation Method of Plasmid DNA-Loaded, Extracellular Vesicles-Mimicking Lipid Nanoparticles Based on Nucleic Acids Dilution-Induced Assembly

**DOI:** 10.3390/cells13141183

**Published:** 2024-07-11

**Authors:** Kazuya Okami, Shintaro Fumoto, Mana Yamashita, Moe Nakashima, Hirotaka Miyamoto, Shigeru Kawakami, Koyo Nishida

**Affiliations:** Graduate School of Biomedical Sciences, Nagasaki University, 1-7-1 Sakamoto, Nagasaki 852-8501, Japan; bb55321402@ms.nagasaki-u.ac.jp (K.O.); hmiyamoto@nagasaki-u.ac.jp (H.M.); skawakam@nagasaki-u.ac.jp (S.K.); koyo-n@nagasaki-u.ac.jp (K.N.)

**Keywords:** extracellular vesicles, drug delivery system, lipid nanoparticles, microfluidic device, plasmid DNA, HepG2 cells, gene delivery

## Abstract

We propose a nucleic acids dilution-induced assembly (NADIA) method for the preparation of lipid nanoparticles. In the conventional method, water-soluble polymers such as nucleic acids and proteins are mixed in the aqueous phase. In contrast, the NADIA method, in which self-assembly is triggered upon dilution, requires dispersion in an alcohol phase without precipitation. We then investigated several alcohols and discovered that propylene glycol combined with sodium chloride enabled the dispersion of plasmid DNA and protamine sulfate in the alcohol phase. The streamlined characteristics of the NADIA method enable the preparation of extracellular vesicles-mimicking lipid nanoparticles (ELNPs). Among the mixing methods using a micropipette, a syringe pump, and a microfluidic device, the lattermost was the best for decreasing batch-to-batch differences in size, polydispersity index, and transfection efficiency in HepG2 cells. Although ELNPs possessed negative ζ-potentials and did not have surface antigens, their transfection efficiency was comparable to that of cationic lipoplexes. We observed that lipid raft-mediated endocytosis and macropinocytosis contributed to the transfection of ELNPs. Our strategy may overcome the hurdles linked to supply and quality owing to the low abundance and heterogeneity in cell-based extracellular vesicles production, making it a reliable and scalable method for the pharmaceutical manufacture of such complex formulations.

## 1. Introduction

Extracellular vesicles (EVs) such as exosomes are released from most cell types and are potent mediators of cell-to-cell communication [1,2]. EVs contain proteins, nucleic acids, and lipids from donor cells and act as natural communication systems which are crucial in biological dynamics such as cancer metastasis [3]. The use of EVs to encapsulate therapeutic agents is gaining momentum and some formulations have reached the clinical development stage [4]. EVs have several benefits as drug carriers, including their biological origin, safety, and tissue selectivity [5]. EVs have the potential to effectively treat diseases, such as myocardial infarction [6], brain ischemia-reperfusion injury [7], acute lung injury [8], and skeletal muscle injury [9]. Exogenous nucleic acids can also be incorporated into EVs by fusing EVs with pre-prepared nucleic acids delivery systems [10], cell extrusion [11], electroporation [12], and ultrasound [13], thereby improving the applicability of EV therapeutics. However, persistent challenges in EV supply and heterogeneity must be addressed [14]. The cell-based production of EVs is costly and requires large-scale and high-quality manufacture because of their heterogeneity and low abundance [15]. Drug delivery systems that mimic the lipid composition of EVs [16] can theoretically solve these problems. However, the encapsulation efficiency of nucleic acids into EVs, heterogeneity, and mass production of EVs mimics remain to be improved [17].

Lipid nanoparticles (LNPs) encapsulating nucleic acids, such as siRNA, mRNA, and plasmid DNA, have been developed [18,19,20]. The ethanol (EtOH) injection method combined with microfluidics resolves the problems of heterogeneity and mass production [21]. We hypothesized that EV-mimicking lipid nanoparticles (ELNPs) could be prepared using the EtOH injection method. However, the anionic nature of lipids within EVs hinders nucleic acids encapsulation in vesicles via electrostatic repulsion. EVs contain histones which may contribute to nucleic acids encapsulation [22]; however, histone purification remains challenging [23], making their use for gene delivery uncommon. Protamine sulfate is a histone alternative in gene delivery [24] because of its established clinical application [25]. In this study, we designed LNPs containing plasmid DNA and protamine sulfate coated with a lipid membrane that mimics the lipid composition of EVs. The lipid composition of the EVs was based on the composition reported in a lipidomics study [26]. In conventional methods, water-soluble protamine sulfate and plasmid DNA are added to the aqueous phase because these materials precipitate in EtOH [27,28]. The mixture is then combined with a lipid alcohol solution to form LNPs. The multi-step process for LNP formulation is complex and faces difficulties in size control and reproducibility. To address these issues, we hypothesized that dispersing all components, including lipids, protamine sulfate, and plasmid DNA in the alcohol phase, would realize the one-step formation of LNPs after injecting the alcohol phase into the aqueous phase. There may be suitable alcohols to disperse protamine sulfate and plasmid DNA without precipitation and complexation due to ineffective hydration in alcoholic solutions. Diluting the alcohol phase with the aqueous phase would trigger the self-assembly of cationic protamine sulfate, anionic plasmid DNA, and lipids, and was thus termed the nucleic acids dilution-induced assembly (NADIA) method.

In this study, we tested several alcohols, including propylene glycol (PG), as a novel approach to simultaneously dispersing water-soluble polymers without precipitation in the alcohol phase. We subsequently prepared complex ELNPs composed of plasmid DNA, protamine sulfate, and major lipids contained in EVs through one-step alcohol injection. We conducted preparations using a micropipette, a syringe pump, and a microfluidic device. We then assessed batch-to-batch variability in physicochemical properties and transfection efficiency in human hepatocellular carcinoma HepG2 cells, thereby demonstrating the effectiveness of the innovative preparation technique and the quality of the resulting nanoparticles. We also elucidated the transfection mechanisms of ELNPs by using endocytosis inhibitors. Typically, EVs are taken up by recipient cells through recognition based on surface antigens [29,30,31]. However, our ELNPs lacked surface antigens during their formulation design. Such artificial systems will contribute to the understanding of the function of EVs under normal and pathological conditions and lead to the discovery of novel drug targets.

## 2. Materials and Methods

### 2.1. Materials

D-(+)-Glucose, ethidium bromide, 1-propanol (1-Pro), 2-propanol (2-Pro), EtOH, Triton X-100, dextran sodium sulfate, and sodium chloride (NaCl) were purchased from Nacalai Tesque (Kyoto, Japan). *N*-octanoyl-sphingosine-1-{succinyl[methoxy(polyethylene glycol)2000]} (PEG-Cer) and 1,2-dioleoyl-3-trimethylammonium-propane (DOTAP) methyl sulfate salt were obtained from Avanti Polar Lipids (Alabaster, AL, USA). Protamine sulfate, bovine serum albumin, and methyl-β-cyclodextrin (MβCD) were purchased from Sigma-Aldrich (St. Louis, MO, USA). PG, glycerol (Gly), penicillin-streptomycin solution (×100), fish oil-derived sphingomyelin (SM), and cholesterol (Chol) were purchased from the FUJIFILM Wako Pure Chemical Corporation (Osaka, Japan). 1,2-Dioleoyl-*sn*-glycero-3-phosphocholine (DOPC), 1,2-dioleoyl-*sn*-glycero-3-phosphoethanolamine (DOPE), 1,2-dioleoyl-*sn*-glycero-3-phospho-L-serine, and sodium salt (DOPS-Na) were purchased from the NOF Corporation (Tokyo, Japan). Agarose S was purchased from NIPPON GENE Co., Ltd. (Toyama, Japan). Chlorpromazine hydrochloride (CPZ) was obtained from Tokyo Chemical Industry Co., Ltd. (Tokyo, Japan). Ethyl-isopropyl-amiloride (EIPA) was purchased from R&D Systems (Minneapolis, MN, USA). All cell culture media were purchased from Thermo Fisher Scientific (Waltham, MA, USA).

### 2.2. Plasmid DNA

The plasmid DNA pcDNA3/GL vector encoding secreted *Gaussia princeps* luciferase (Gluc) under the CMV promoter was purchased from Lux Biotechnology Ltd. (Edinburgh, UK). Following the amplification process in the DH5α strain of *Escherichia coli*, purification was performed using the EndoFree Plasmid Giga Kit (QIAGEN GmbH, Hilden, Germany). Subsequently, the plasmid DNA was dissolved in sterile and filtered ultrapure water. The dissolved plasmid DNA was preserved at −20 °C until further application. The preserved plasmid DNA was appropriately diluted to achieve the specified concentration, following the experimental requirements.

### 2.3. Cell Culture

HepG2 cells from RIKEN (Tokyo, Japan) were cultured in Dulbecco’s modified Eagle’s medium (DMEM) supplemented with 10% fetal bovine serum (FBS), penicillin G (100 units/mL), and streptomycin (100 μg/mL) under standard conditions in a humidified atmosphere with 5% CO_2_ at 37 °C.

### 2.4. Differential Interference Microscopy (DIC) Observation

Solutions containing 1-Pro, 2-Pro, EtOH, PG, Gly, and water, each with or without NaCl (140 mM), were used as the solvents. The plasmid DNA (3 mg/mL in water) was diluted to a final concentration of 20 µg/mL in each solvent. Protamine sulfate (2 mg/mL in water) was diluted to a final concentration of 1 mg/mL in each solvent. To evaluate the dispersibility, a plasmid DNA solution was added to protamine sulfate. The mixtures were incubated for 10 min, degassed for 5 min at 25 °C (Bransonic^®^ CPX1800H-J; Branson Ultrasonics Corporation, Brookfield, CT, USA), and observed using an inverted microscope (Axio Vert.A1 with Plan-Apochromat 20×/0.8 M27 objective lens; Carl Zeiss Microscopy GmbH, Oberkochen, Germany).

### 2.5. Turbidity Measurement

After adding protamine sulfate (1 mg/mL) and plasmid DNA (3 mg/mL) to the various solvents, each mixture underwent more than 30 pipetting cycles. Absorbance at 660 nm was measured using an ultraviolet–visible spectrometer (UV-1850; Shimadzu Corporation, Kyoto, Japan).

### 2.6. Agarose Gel Electrophoresis

Agarose gel electrophoresis was performed to assess the presence of the naked plasmid DNA. One percent agarose gel was prepared using a TAE buffer (40 mM Tris-HCl, 40 mM acetic acid, and 1 mM EDTA). A mixture of prepared samples (naked plasmid DNA or protamine sulfate/plasmid DNA complex) and loading dye (complex/loading dye = 5/1; *v*/*v*) (18 µL) was applied to a 1% agarose gel. Electrophoresis was conducted in the TAE buffer using the electrophoresis device Mupid^®^ (ADVANCE, Tokyo, Japan) at 100 V for 30 min. After electrophoresis, the gel was gently shaken for 20 min in a 0.5 µg/mL ethidium bromide solution (100 mL) and then observed under UV light. Protamine sulfate and plasmid DNA were mixed at various N/P ratios according to the following calculation method: N/P ratio = (nitrogen molecules in protamine sulfate)/(a phosphate residue of plasmid DNA). The N/P ratio of 1 corresponds to a mass ratio of 0.594 (protamine sulfate/plasmid DNA).

### 2.7. Preparation of ELNPs

Considering the lipid composition of the EVs [26], the lipid ratio of ELNPs was set as DOPC:DOPE:DOPS:SM:Chol = 18:7:13:17:45. Additionally, to improve the dispersion, PEG-Cer (1 mol % of total lipids) was included. Before mixing protamine sulfate and plasmid DNA with lipids in the alcohol phase, protamine sulfate and plasmid DNA were separately dispersed in PG containing 140 mM NaCl. As the first step in preparing the alcohol phase, PG, protamine sulfate (0.2 mg/mL in PG/water (93/7) with 140 mM NaCl) and plasmid DNA (0.3 mg/mL in PG/water (87/13) with 140 mM NaCl) were mixed well at a volume ratio of 46.7:30.6:22.7. Subsequently, the lipids were added to a mixture containing 18 µg protamine sulfate and 20 µg plasmid DNA in the following order: Chol (10 mg/mL, EtOH), DOPC (10 mg/mL, EtOH), DOPE (5 mg/mL, EtOH), SM (4 mg/mL, EtOH), PEG-Cer (2 mg/mL, EtOH), and DOPS-Na (1.5 mg/mL, EtOH/PG = 2/1). EtOH and NaCl (280 mg/mL, water) were added to the mixture to adjust the volume ratio of PG (50%) and the concentration of NaCl (140 mM), respectively. The lipid amount was 0.5, 1.0, and 1.5 μmol/10 μg of plasmid DNA. The aqueous phase was composed of an acetate buffer (100 mM, pH 4.4). The aqueous phase/alcohol phase ratios were set at 1, 2, and 3 (*v*/*v*). In the micropipette mixing method (conditions M1–M18), using a micropipette (PIPETMAN^®^; GILSON Inc., Villiers-le-Bel, France), the alcohol phase (0.3 mL) was injected into the aqueous phase (0.3, 0.6, or 0.9 mL) with an injection speed of approximately 1.8 or 0.18 mL/min. The mixture was stirred at 500 rpm for 10 min using a magnetic stirrer (RS-1DN; AS ONE, Osaka, Japan). After mixing, the solution was diluted with PBS(−) (9.4, 9.1, and 8.8 mL, according to the aqueous phase/alcohol phase ratio), followed by ultrafiltration (2000× *g*, 20 min, 25 °C, Amicon^®^Ultra-15; Merck KGaA, Darmstadt, Germany), and then diluted again with 10 mL PBS(−). Ultrafiltration was performed again, and the final recovery was performed with 1 mL of PBS(−). For the preparation using a syringe pump (conditions S1–S9), the composition of the alcohol and aqueous phases was the same as that for the micropipette mixing method. The injection rates were set at 1, 6.5, and 12 mL/h and the aqueous phase/alcohol phase ratios were adjusted to 1, 2, and 3 (*v*/*v*). Stirring at 500 rpm for 10 min was applied during the mixing of the alcohol and aqueous phases. Dilution, ultrafiltration, and recovery after injection were the same as those in the micropipette mixing method. For the preparation using a microfluidic device (NanoAssemblr^®^; Precision NanoSystems, Vancouver, BC, Canada) (conditions MF1–MF9), the composition of the alcohol and aqueous phases was the same as that for the micropipette mixing method. The total flow rates (TFRs) were set at 1, 6.5, and 12 mL/min, and the flow rate ratios (FRRs) were set at 1, 2, and 3. The operation after mixing with the microfluidic device was the same as that for the micropipette mixing method.

To compare the preparation methods, the thin lipid film hydration method (Bangham method) was used. The lipid mixture was composed of DOPC, DOPE, DOPS-Na, SM, and Chol at a molar ratio of 18:7:13:17:45. The lipids were dissolved in chloroform and the solvent was subsequently removed under vacuum using a rotary evaporator to produce a dried thin lipid film. The lipid film was hydrated with an acetate buffer (100 mM, pH 4.4) to achieve a final lipid concentration of 2 mg/mL. The mixture was thoroughly vortexed to obtain a lipid suspension. The lipid suspension was mixed at a 1:1 volume ratio with a protamine sulfate/plasmid DNA suspension at an N/P ratio of 1.8, which was also prepared in an acetate buffer (100 mM, pH 4.4). The resulting mixture was incubated at 37 °C for 1 h to allow complex formation. Following incubation, the mixture was diluted with PBS(−) and subjected to ultrafiltration (2000× *g*, 20 min, 25°C, Amicon^®^Ultra-15). Dilution and ultrafiltration were repeated, and the final product was recovered in 1 mL of PBS(−).

The conventional ethanol injection method was also employed. In this method, lipids were added to the alcohol phase, and plasmid DNA/protamine sulfate complexes were added to the aqueous phase. The lipids were added to EtOH in the following order: Chol (10 mg/mL, EtOH), DOPC (10 mg/mL, EtOH), DOPE (5 mg/mL, EtOH), SM (4 mg/mL, EtOH), PEG-Cer (2 mg/mL, EtOH), and DOPS-Na (1.5 mg/mL, EtOH/PG = 2/1). The lipid ratio was 1.0 μmol/10 μg plasmid DNA. The aqueous phase was composed of an acetate buffer (100 mM, pH 4.4) containing 18 µg protamine sulfate and 20 µg plasmid DNA. The aqueous phase/alcohol phase ratio was set at 3 (*v*/*v*). In the conventional ethanol injection, using a micropipette (PIPETMAN^®^), the alcohol phase (0.3 mL) was injected into the aqueous phase (0.9 mL) at an injection speed of approximately 0.18 mL/min. The mixture was stirred at 500 rpm for 10 min using a magnetic stirrer (RS-1D). After mixing, the solution was diluted with PBS(−) 8.8 mL, according to the aqueous phase/alcohol phase ratio, followed by ultrafiltration (2000× *g*, 20 min, 25 °C, Amicon^®^Ultra-15). Dilution and ultrafiltration were repeated, and the final product was recovered in 1 mL of PBS(−).

### 2.8. Preparation of Lipoplexes

The method for preparing cationic lipoplexes was based on that of another study [32]. Briefly, a methanolic solution containing DOTAP methyl sulfate salt and cholesterol at a molar ratio of 1:1 was subjected to vacuum desiccation using a rotary evaporator. The obtained thin lipid film was then hydrated with sterile 5% glucose (8 mg total lipids/mL) and vortexed to yield cationic liposomes. Liposomes were extruded 11 times through a polycarbonate membrane filter (100 nm pore size) using a Mini-Extruder (Avanti Polar Lipids). For lipoplex preparation, plasmid DNA in 5% glucose was added to an equal volume of cationic liposomes at a charge ratio of 2.3 and incubated for 30 min at 37 °C. 

### 2.9. Particle Size and Zeta Potential Measurement

The ENLPs and lipoplexes were diluted with PBS(−) (pH 7.4) and 5% glucose (pH 5.8), respectively, to achieve a plasmid DNA concentration of 1 μg/800 μL. Particle size and zeta potential were measured using a Zetasizer Pro (Malvern Instruments, Malvern, UK).

### 2.10. Evaluation of Transfection Efficiency

HepG2 cells were seeded in a 96-well plate at a density of 1 × 10^4^ cells/well and cultured for 24 h. The medium was replaced with fresh 10% FBS/DMEM, and lipoplexes and ELNPs (200 ng/well pcDNA3/GL) were added, followed by incubation for 2 h. Subsequently, the cells were washed with PBS(−) (pH 7.4) and further incubated in 10% FBS/DMEM for an additional 22 h at 37 °C. Transfection efficiency was evaluated by mixing 4 μL of culture medium with 20 μL of a *Renilla* luciferase assay system containing Gluc substrate coelenterazine (Promega, Madison, WI, USA), and bioluminescence levels were measured using a luminometer (Lumat LB 9507; Berthold Technologies, Bad Wildbad, Germany). Luciferase activity was expressed as relative light units (RLU)/mL of medium.

### 2.11. Endocytosis Inhibition

For endocytosis inhibition experiments, stock solutions of CPZ (clathrin-mediated endocytosis inhibitor), MβCD (lipid raft-mediated endocytosis inhibitor), and EIPA (macropinocytosis inhibitor) were prepared at concentrations of 5, 10, and 5 mg/mL, respectively, in dimethyl sulfoxide (DMSO) (CPZ, EIPA) or water (MβCD). The stock solutions were subsequently diluted to final concentrations of 5–20 μM, 5–20 mM, and 5–15 μM, respectively, in DMEM (10% FBS). The inhibitor groups were pre-incubated with media containing inhibitors for 30 min before transfection. Inhibitor concentrations were determined based on the pretest results. After applying the endocytosis inhibitor, transfection was performed as described in Section 2.10. 

### 2.12. Evaluation of Encapsulation Efficiency

The encapsulation efficiency of the samples was determined using the QuantiFluor^®^ ONE dsDNA System and Quantus™ Fluorometer (Promega, Madison, WI, USA). The encapsulation efficiency was calculated using the following equation:

$$Encapsulation\;efficiency\;(\%)=\frac{Total\;DNA-free\;DNA}{Total\;DNA}\times 100$$

To measure free DNA, samples were incubated with PBS at a 1:1 (*v*/*v*) ratio at 37 °C for 10 min. Total DNA was determined by mixing the samples with PBS containing 0.25% (*w*/*w*) Triton X and 0.008% (*w*/*v*) dextran sulfate at a 1:1 (*v*/*v*) ratio, followed by incubation at 37 °C for 10 min. All subsequent steps were performed according to the manufacturer’s protocol.

### 2.13. Statistical Analysis

Statistical comparisons were conducted using a one-way analysis of variance, followed by Dunnett’s post hoc test for multiple comparisons against the control group. Statistical significance was set at * *p* < 0.05, ** *p* < 0.01, and *** *p* < 0.001.

## 3. Results

### 3.1. Suitable Alcohol Phases to Disperse Plasmid DNA and Protamine Sulfate

#### 3.1.1. Alcohol Phases for Dispersing Plasmid DNA

Protamine sulfate and plasmid DNA can disperse independently when placed in water. However, when combined to form a complex, the protamine/plasmid complex aggregated in water (Figure S1). Therefore, we attempted to disperse protamine sulfate and plasmid DNA in alcohols without complexation.

**Figure 1:**
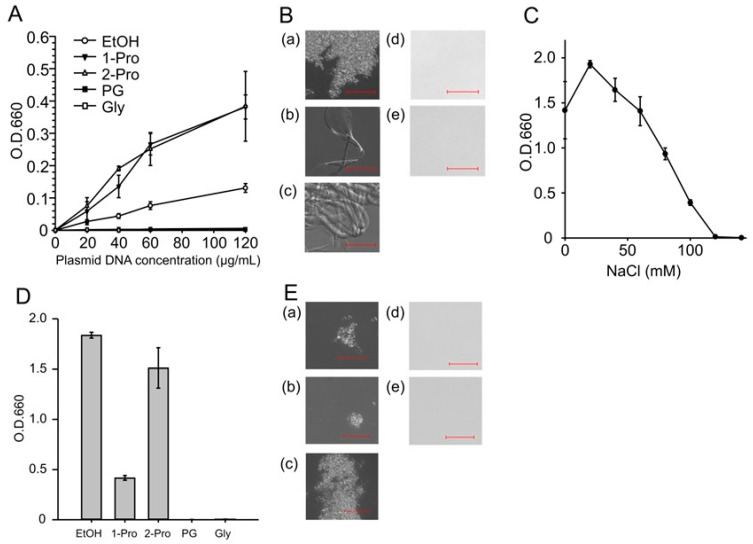
Microscopic observation and turbidity measurement for dispersing plasmid DNA and protamine sulfate without precipitation. (**A**) Turbidity (optical density, O.D. 660) of plasmid DNA dispersions in various alcohols. 1-Pro, 1-propanol; 2-Pro, 2-propanol; EtOH, ethanol; PG, propylene glycol; Gly, glycerol. Each dot represents the mean ± standard deviation (SD) of three experiments. (**B**) Differential interference microscopy (DIC) images of plasmid aggregates in various alcohols. (**a**) 1-Pro, (**b**) 2-Pro, (**c**) EtOH, (**d**) PG, (**e**) Gly. Scale bar, 50 μm. (**C**) Turbidity of protamine sulfate dispersions (1 mg/mL) in PG with NaCl (0–140 mM). Each dot represents the mean ± SD of three experiments. (**D**) Turbidity of protamine sulfate dispersions in various alcohols containing 140 mM NaCl. Each bar represents the mean ± SD of three experiments. (**E**) DIC images of protamine sulfate aggregates in various alcohols containing NaCl (140 mM). (**a**) 1-Pro, (**b**) 2-Pro, (**c**) EtOH, (**d**) PG, (**e**) Gly. Scale bar, 50 μm.

First, we assessed the dispersibility of plasmid DNA in different alcohols based on turbidity (Optical Density (O.D.) 660) and DIC microscopy. EtOH, 1-Pro, and 2-Pro enhanced the turbidity with increasing concentrations, whereas PG and Gly did not (Figure 1A). Furthermore, visible plasmid DNA precipitates were observed in 1-Pro, 2-Pro, and EtOH (Figure 1B). In contrast, no precipitation of plasmid DNA was observed in PG and Gly.

#### 3.1.2. Alcohol Phase for Dispersing Protamine Sulfate

The precipitation of protamine sulfate (1 mg/mL) was investigated under various solvent conditions. We observed the precipitation of protamine sulfate in preliminary experiments using 1-Pro, 2-Pro, EtOH, and PG as the solvents. However, no precipitation was observed in Gly or water (Figure S1A and Figure S2A). In the turbidity measurement of protamine sulfate in alcohols, only Gly exhibited a low O.D.660 value (Figure S2B). In a preliminary experiment employing the NADIA method for the preparation of ELNPs using Gly, the particle sizes were greater than 150 nm and varied widely (Figure S2C and Table S1). Additionally, the wrong particle homogeneities (polydispersity index (PDI) > 0.3) were observed (Figure S2C and Table S1). Therefore, we investigated the application of NaCl in the PG phase as a more appropriate method for preparing ELNPs. This adjustment significantly reduced the absorbance to below 0.1, especially when the NaCl concentration was above 120 mM (Figure 1C). Furthermore, we added NaCl to each alcohol phase at a concentration of 140 mM, and the absorbance of PG and Gly was less than 0.01 (Figure 1D). DIC microscopy corroborated these findings and revealed the absence of precipitates in PG and Gly at an NaCl concentration of 140 mM (Figure 1E). These observations highlight that the solvent composition, especially the judicious addition of NaCl, plays a fundamental role in mitigating the precipitation tendency and enhancing the dispersibility of protamine sulfate in the PG phase.

### 3.2. Formation of Protamine Sulfate and Plasmid DNA Complexes in an Aqueous Condition

**Figure 2:**
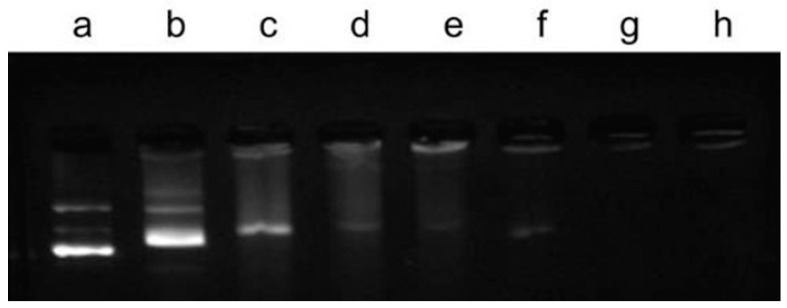
Agarose gel retardation assay of protamine sulfate and plasmid DNA mixtures. Plasmid DNA (100 ng) was added to each well. (**a**) Free naked plasmid DNA. (**b**–**h**) Mixtures of protamine sulfate and plasmid DNA in water at various N/P ratios (b, 0.5; c, 1.0; d, 1.2; e, 1.4; f, 1.6; g, 1.8; h, 2.0).

We optimized the N/P ratio governing complex formation between protamine sulfate and plasmid DNA under aqueous conditions. Above an N/P ratio of 1.8, no naked plasmid DNA bands were observed (Figure 2), indicating complex formation.

### 3.3. Possible Alcohol Phase Allowing for the Simultaneous Dispersion of Protamine Sulfate and Plasmid DNA

**Figure 3:**
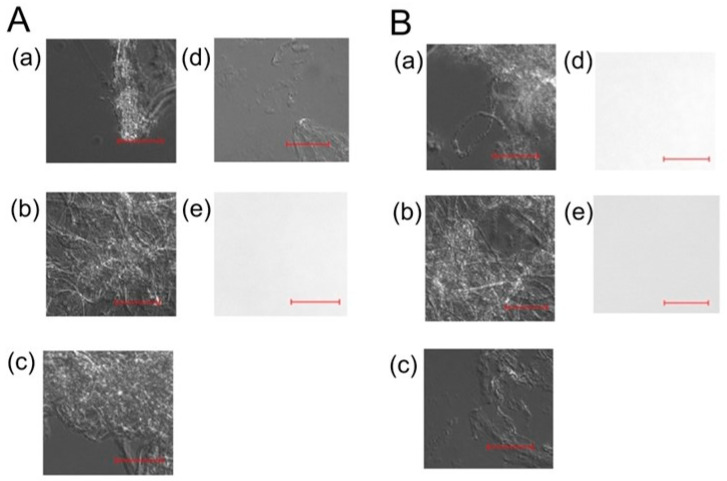
Microscopic images of plasmid DNA and protamine sulfate precipitates in various alcohols. (**A**) Differential interference microscopy (DIC) images of plasmid DNA and protamine sulfate precipitation in the presence of various alcohols without NaCl. (**a**) 1-Pro, 1-propanol; (**b**) 2-Pro, 2-propanol; (**c**) EtOH, ethanol; (**d**) PG, propylene glycol; (**e**) Gly, glycerol. Scale bar, 50 μm. (**B**) DIC images of plasmid DNA and protamine sulfate precipitation in various solvents containing NaCl (140 mM). (**a**) 1-Pro, (**b**) 2-Pro, (**c**) EtOH, (**d**) PG, (**e**) Gly. Scale bar: 50 μm.

We tested the possibility of simultaneously mixing protamine sulfate and plasmid DNA in alcohol phases without precipitation. In the alcohol phases (1-Pro, 2-Pro, EtOH, and PG) without NaCl, the precipitates were observed by microscopy (Figure 3A). The addition of 140 mM NaCl to PG prevented precipitation (Figure 3B). Furthermore, we tested whether the precipitation of protamine sulfate and plasmid DNA occurred in the EtOH and PG mixed solutions (140 mM NaCl) (Figure S3A). The aggregation of protamine sulfate and plasmid DNA was greatly suppressed in the mixture of EtOH and PG, and PG ratios exceeding 20% were crucial. Considering the addition of the lipid EtOH solution for subsequent experiments, the alcohol phase was set to contain EtOH and PG (1/1, *v*/*v*) with 140 mM NaCl. The agarose retardation assay revealed that plasmid DNA did not form complexes with protamine sulfate in the alcohol phase, whereas we observed the complex formation of plasmid DNA with protamine sulfate after dilution with the aqueous phase (Figure S3B).

### 3.4. Preparation of ELNPs through the Micropipette Mixing NADIA Method

**Figure 4:**
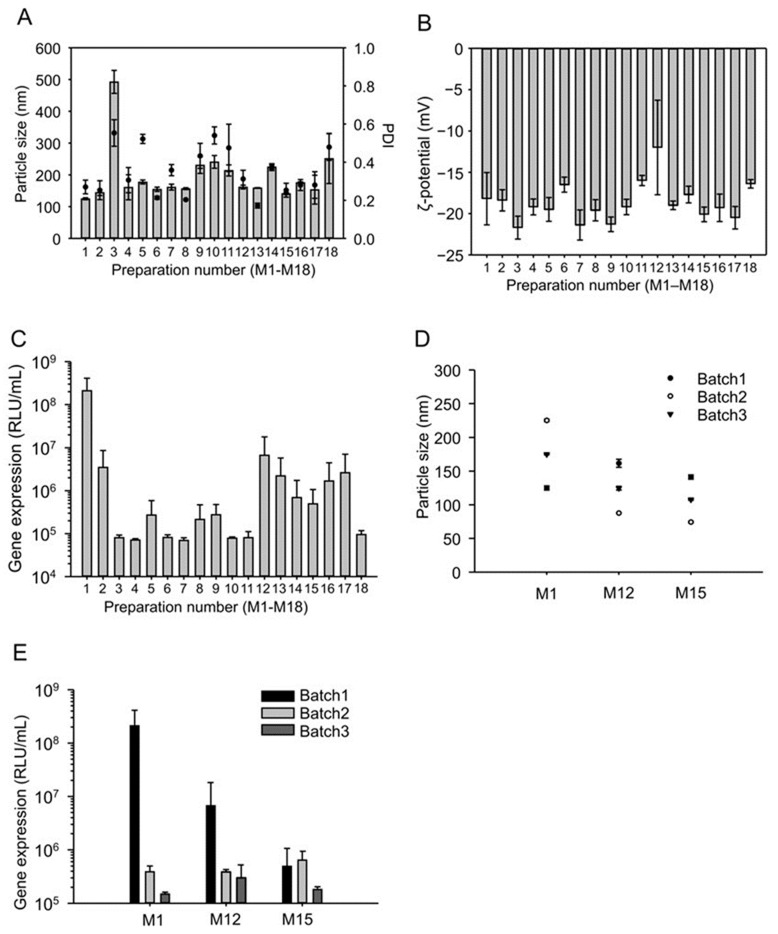
Physicochemical properties and transfection efficiencies of ELNPs prepared by micropipette mixing. (**A**) Particle size (bars) and polydispersity index (PDI) (dots). (**B**) ζ-potential. Each bar and dot represent the mean ± standard deviation (SD) of three replicates. (**C**) Transfection efficiency. Each bar represents the mean + SD of three replicates. (**D**) Batch-to-batch differences in particle size. Each dot represents the mean ± SD of three replicates. (**E**) Batch-to-batch differences in transfection efficiency. Each bar represents the mean ± SD of three replicates. ELNPs were prepared thrice and designated as Batch1, Batch2, and Batch3. ELNPs, EV-mimicking lipid nanoparticles; RLU, relative light units.

We tested the feasibility of preparing LNPs that mimicked EV function based on micropipette mixing using the NADIA method. In a preliminary experiment, we confirmed the absence of aggregation after adding lipids to the alcohol phase (Figure S4). Subsequently, we analyzed the lipid/plasmid DNA ratio (µmol/10 µg), aqueous phase/alcohol phase ratio, and alcohol phase injection rate (Table 1 and Figure S5A). This assessment aimed to understand the effects of these factors on the physicochemical characteristics of the particles and their transfection efficiency in HepG2 cells (Figure 4A–C). We also tested batch-to-batch variations using micropipette mixing, based on the NADIA method (Figure 4D,E). To determine the ideal lipid/plasmid DNA ratio, we systematically explored different ratios: 0.5 μmol/10 μg plasmid DNA for M1–M6, 1.0 μmol/10 μg plasmid DNA for M7–M12, and 1.5 μmol/10 μg plasmid DNA for M13–M18. We conducted a thorough investigation, taking both physicochemical properties and gene expression efficiency into account. We identified M1 (with the highest gene expression level, 2.1 × 10^8^ RLU/mL, and the smallest particle size, 124.9 nm, among M1–M6), M12 (with the highest gene expression level, 6.7 × 10^6^ RLU/mL, and small particle size, 161.6 nm, among M7–M12), and M15 (with a high gene expression level, 5.0 × 10^5^ RLU/mL, and the smallest particle size, 141.1 nm, among M13–M18) as the optimal conditions. ELNPs were prepared thrice (Batches 1–3) for M1, M12, and M15. There were large batch-to-batch differences in particle size (Figure 4D) and transfection efficiency (Figure 4E).

### 3.5. Preparation of ELNPs through the Syringe Pump NADIA Method

**Figure 5:**
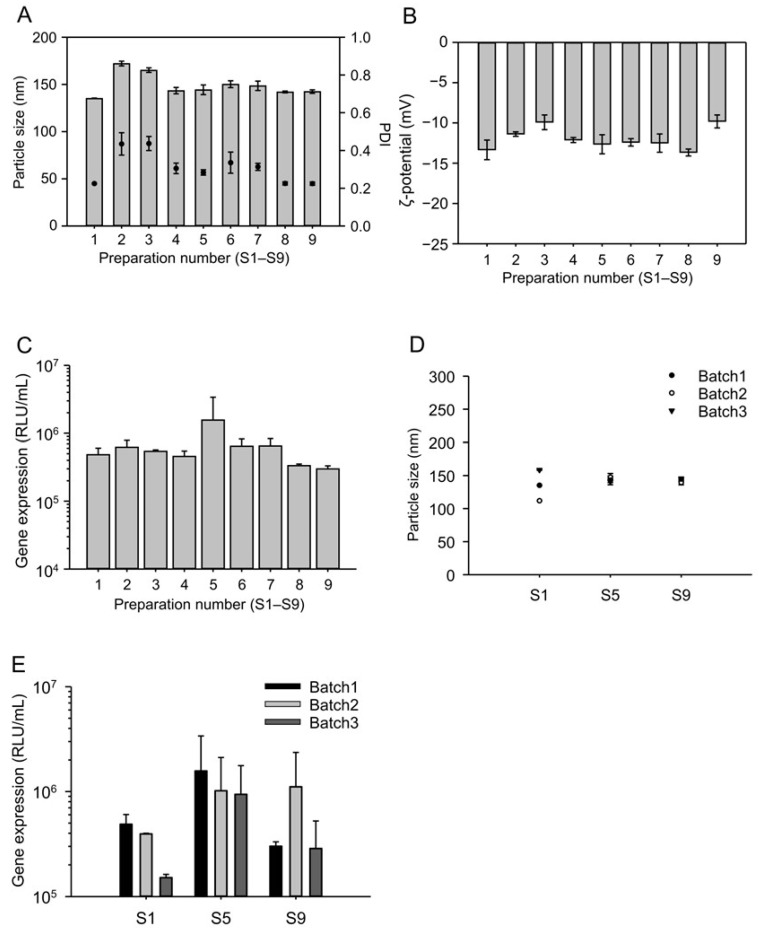
Physicochemical properties and transfection efficiencies of ELNPs prepared using the syringe pump NADIA method. (**A**) Particle size (bars) and polydispersity index (PDI) (dots). (**B**) ζ-potential. Each bar and dot represent the mean ± standard deviation (SD) of three replicates. (**C**) Transfection efficiencies. Each bar represents the mean + SD of three replicates. (**D**) Batch-to-batch differences for particle size. Each dot represents the mean ± SD of three replicates. (**E**) Batch-to-batch differences for transfection efficiency. Each bar represents the mean + SD of three replicates. ELNPs were prepared thrice and designated as Batch1, Batch2, and Batch3. ELNPs, EV-mimicking lipid nanoparticles; NADIA, nucleic acids dilution-induced assembly; RLU, relative light units.

We demonstrated that the preparation of ELNPs using the micropipette mixing NADIA method resulted in variations in both physicochemical properties and gene expression efficiency. To address this issue, we integrated the NADIA method with controlled injection using a syringe pump. ELNPs were prepared by injecting the alcohol phase from a 1 mL syringe into the aqueous phase at a constant rate (Table 2 and Figure S5B). The lipid/plasmid DNA ratio was set at 1 µmol/10 µg of plasmid DNA. Additionally, we investigated the aqueous phase/alcohol phase ratios (1, 2, and 3) and injection rates (1, 6.5, and 12 mL/h). We identified conditions characterized by the smallest particle size of 135.2 nm (S1), highest gene expression efficiency of 1.5 × 10^6^ RLU/mL (S5), and high formulation homogeneity (PDI 0.22) (S9) (Figure 5A–C). We rigorously tested the reproducibility under these conditions (S1, S5, and S9) and evaluated both physicochemical properties and gene expression efficiency. The application of the syringe pump reduced variations in particle size and gene expression efficiency (Figure 5D,E).

### 3.6. Preparation of ELNPs through the Microfluidic NADIA Method

We investigated the feasibility of preparing lipid nanoparticles using NADIA with a microfluidic device. Throughout this experiment, we referred to the particle preparation conditions from both the micropipette mixing and syringe pump NADIA methods.

**Figure 6:**
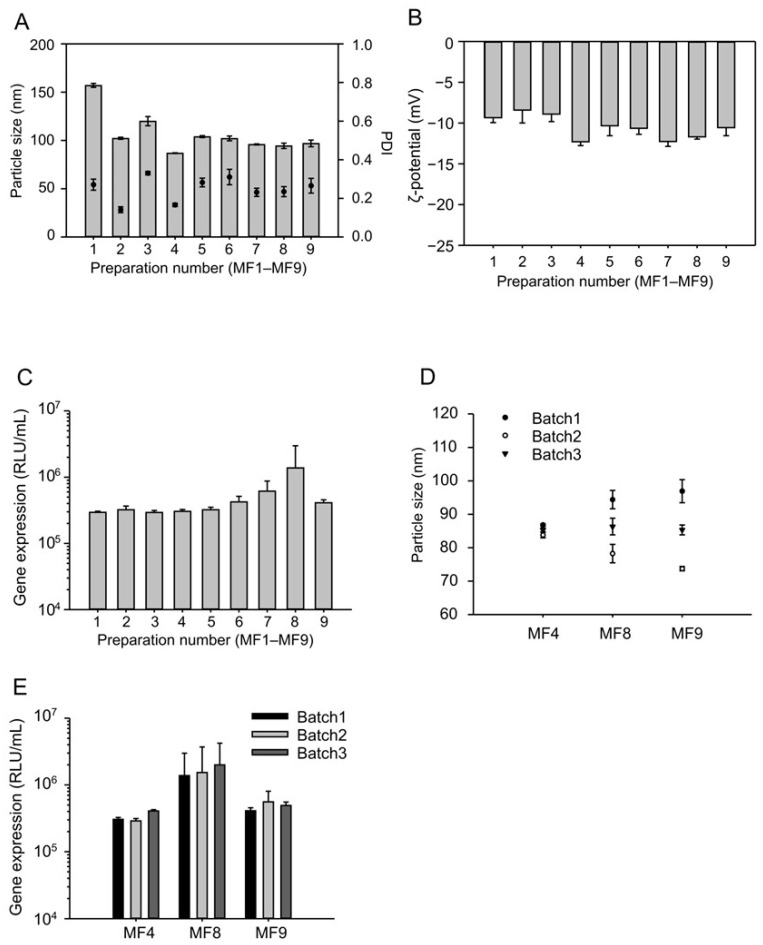
Physicochemical properties and transfection efficiencies of ELNPs prepared using the microfluidic NADIA method. (**A**) Particle size (bars) and polydispersity index (PDI) (dots). (**B**) ζ-potential. Each bar and dot represent the mean ± standard deviation (SD) of three replicates. (**C**) Transfection efficiencies. Each bar represents the mean + SD of three replicates. (**D**) Batch-to-batch differences for particle size. Each dot represents the mean ± SD of three replicates. (**E**) Batch-to-batch differences for transfection efficiency. Each bar represents the mean + SD of three replicates. Exosome-mimicking lipid nanoparticles (ELNPs) were prepared thrice and designated as Batch1, Batch2, and Batch3. NADIA, nucleic acids dilution-induced assembly; RLU, relative light units.

The lipid/plasmid DNA ratio was set at 1 µmol/10 µg of plasmid DNA. Subsequently, the aqueous phase/alcohol phase ratio (FRR) and flow rate (TFR) were analyzed (Table 3 and Figure S5C). The results revealed three optimal conditions: MF4 (minimal particle size, 86.8 nm), MF8 (maximal gene expression efficiency, 1.3 × 10^6^ RLU/mL), and MF9 (well-balanced particle size, 96.9 nm, and gene expression efficiency, 4.1 × 10^5^ RLU/mL) (Figure 6A–C). To assess reproducibility under these three conditions (MF4, MF8, and MF9), we evaluated physicochemical properties and gene expression efficiencies (Figure 6D,E). Similar to particle preparation using a syringe pump, reductions in the variability in particle size and gene expression efficiency were confirmed with particle preparation using a microfluidic device based on the NADIA method.

To evaluate the reproducibility, we repeated the preparation nine times for the NADIA micropipette (M1) and NADIA microfluidic mixing (MF8) (Figure S6). Again, the NADIA microfluidic mixing method exhibited high reproducibility.

To evaluate the localization of plasmid DNA in ELNPs, agarose gel electrophoresis was performed (Figure S7). We used the detergent Triton-X100 and dextran sodium sulfate to lyse the lipids of ELNPs and release plasmid DNA from protamine sulfate based on electrostatic interactions, respectively. ELNPs prepared without protamine sulfate contained free plasmid DNA (lane c), indicating insufficient encapsulation. ELNPs containing protamine sulfate showed undetectable plasmid DNA bands (lane d); thus, protamine sulfate was essential for ELNP formation. Treatment of ELNPs with Triton X-100 resulted in the appearance of a free plasmid DNA band (lane f), indicating encapsulation of plasmid DNA in ELNPs. The weak band at the origin of the applied well for the ELNPs treated with dextran sodium sulfate (lane g) may have indicated that part of the plasmid DNA molecules were bound on the surface of ELNPs together with protamine sulfate. Finally, the simultaneous addition of Triton X-100 and sodium dextran sodium sulfate resulted in brighter bands (lane h) compared with lanes f and g. Therefore, most plasmid DNA molecules may be encapsulated in ELNPs.

### 3.7. Comparison of ELNP Preparation Methods

We further compared the characteristics of the ELNPs prepared using the NADIA and other methods (Table S2). The selected methods for preparing ELNPs were the Bangham method and conventional ethanol injection method (adding plasmid DNA and protamine sulfate to an aqueous phase). The particle size and PDI of ELNPs prepared using the Bangham and conventional ethanol injection methods (>200 nm and >0.29, respectively) were much larger than those obtained using the NADIA method. Furthermore, the encapsulation efficiencies of plasmid DNA in ELNPs prepared using the Bangham and conventional ethanol injection methods (<60%) were smaller than those using the NADIA method. The change in the size of ELNPs by the addition of plasmid DNA and protamine sulfate in the Bangham method was larger than that in the alcohol injection method, indicating the usefulness of the latter method.

### 3.8. Essential Endocytic Routes for ELNPs in HepG2 Cells

**Figure 7:**
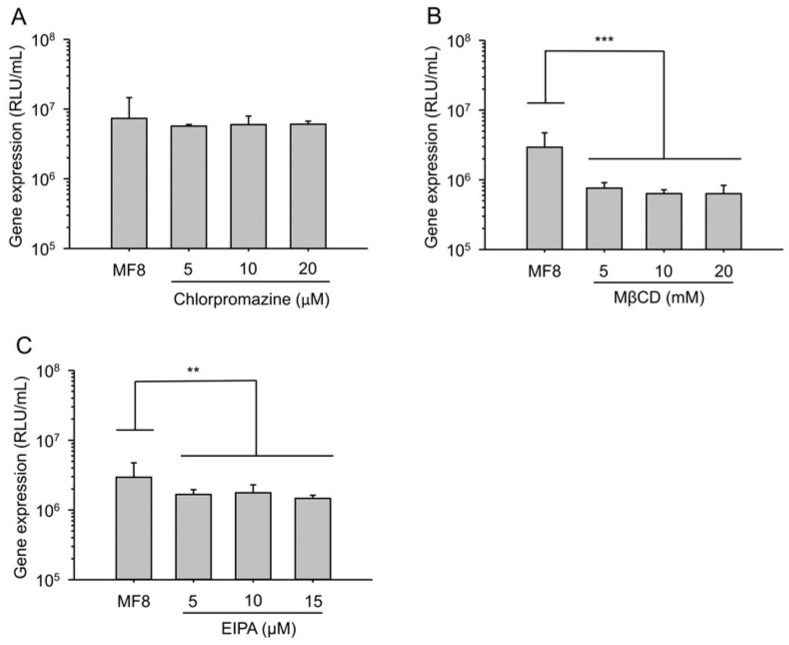
Effects of endocytosis inhibition on the transfection efficiency of ELNPs. Effect of endocytosis inhibition using chlorpromazine (5–20 µM), methyl-β-cyclodextrin (MβCD, 5–20 mM), and ethyl-isopropyl-amiloride (EIPA, 5–15 µM) on the transfection of exosome-mimicking lipid nanoparticle (ELNP) MF8. Each bar represents the mean + standard deviation of three replicates. Statistical analysis was performed using Dunnett’s test (**A**–**C**) ** *p* < 0.01, *** *p* < 0.001 compared to the control group. RLU, relative light units.

ELNPs exhibited lower particle size and ζ-potential and slightly higher transfection efficiency than cationic lipoplexes (Figure S8). To elucidate the mechanisms underlying the high transfection efficiency of ELNPs in HepG2 cells, endocytosis was inhibited before transfection (Figure 7A–C). Clathrin-mediated endocytosis inhibition using CPZ had a minimal effect on transfection efficiency. In contrast, inhibition of lipid raft-mediated endocytosis using MβCD resulted in a significant decrease in the transfection efficiency. Additionally, transfection efficiency significantly decreased when using EIPA, a macropinocytosis inhibitor.

## 4. Discussion

Researchers have developed several methods for preparing liposomes and lipid nanoparticles, including the thin film hydration method (Bangham method) and ethanol injection method [33,34,35]. Although the Bangham method can produce a sufficient quantity of uniform nanoparticles at the laboratory level in combination with several techniques, such as sonication and extrusion, it is unsuitable for industrial applications [36]. In contrast, the ethanol injection method with microfluidics realizes GMP-standard large-scale production [37]. Therefore, in this study, we developed a preparation method based on the ethanol injection method, which is feasible for large-scale production using microfluidics.

To develop ELNPs, we adopted the majority of the lipids found in EVs, specifically phosphatidylcholine, phosphatidylethanolamine, phosphatidylserine, sphingomyelin, and cholesterol [26,38,39]. To formulate ELNPs, PEGylated lipids were added to improve dispersibility. We succeeded in the formulation of ELNPs despite the electric repulsion between the plasmid DNA and anionic lipid phosphatidylserine. Lee and Huang [40] developed the anionic version of lipid–polymer–DNA complexes (LPD II). During the preparation of LPD II, serial mixing of cationic polymers, plasmid DNA, and pre-prepared anionic liposomes is necessary to form complexes. Such serial mixing may not be suitable for large-scale GMP-grade manufacture. In this study, we developed the NADIA method, in which not only lipids but also water-soluble polymers were dispersed in the alcohol phase, enabling the simple one-step formation of LNPs based on alcohol injection. Usually, plasmid DNA and protamine sulfate are dissolved in water due to stabilization by water molecules because they aggregate and precipitate in EtOH [27,28]. Thus, it is necessary to perform serial mixing of a plasmid DNA aqueous solution, a protamine sulfate aqueous solution, and a subsequent lipid alcohol solution for LNP preparation, making LNP formation complex and less reproducible. To develop the NADIA method, it was necessary to investigate suitable conditions to simultaneously disperse plasmid DNA and protamine sulfate in the alcohol phase. We previously demonstrated that PG can disperse plasmid DNA without precipitation [41]. Furthermore, we succeeded in dispersing proteins such as superoxide dismutase and serum albumin in PG [42]. Based on these findings, we hypothesized that plasmid DNA and protamine sulfate could be able to be simultaneously dispersed in the alcohol phase using PG. PG played a significant role in plasmid DNA dispersion (Figure 1). In the case of protamine sulfate, 140 mM NaCl was required for dispersion in PG (Figure 3). We succeeded in the simultaneous dispersion of plasmid DNA and protamine sulfate in the alcohol phase using PG with 140 mM NaCl without complexation (Figure S3), proving our hypothesis. However, the theoretical basis of the NADIA method remains unclear. The dispersion of nucleic acids and proteins in aqueous solutions is caused by hydration based on hydrogen bonds [43].

Ion–dipole interaction [44] may contribute to the sufficient dispersibility of plasmid DNA in PG. Plasmid DNA is a polyanion; therefore, ion–dipole interactions between DNA and solvent molecules are essential for solvation [44]. Monohydric alcohols, such as ethanol, have lower dipole moments than water, whereas polyols (PG and Gly) have higher dipole moments (Table S3). This may explain the differences in the dispersibility of plasmid DNA in alcohols (Figure 1A,B). In contrast, PG was insufficient to disperse the polycation protamine sulfate, whereas Gly dispersed it well (Figure S2). The order of dipole moments is water < PG < Gly; therefore, the ion–dipole interaction alone was insufficient to explain the difference in the dispersibility of protamine molecules in these solvents. The dielectric constants of these solvents (Table S3) may explain the dispersibility because PG has a lower dielectric constant than Gly. A decrease in the dielectric constant due to the addition of ethanol to water enhances the electrostatic interaction between DNA and counterions and subsequently induces complexation [45]. Such effects of counterions form the basis for Manning’s counterion condensation theory [46]. For the protamine used in this study, the counterion was a sulfate ion. The dielectric constant decreased after the addition of PG to the protamine sulfate aqueous solution, and the electrostatic interaction between the protamine and sulfate ions likely became strong. Thereafter, the electrostatic repulsion among the protamine molecules may have weakened, possibly explaining the precipitation of protamine. According to the Hofmeister series, the sulfate ion is a kosmotrope and is more hydrophilic than the chaotropic chloride ion [47,48]. Considering thermodynamics, the Gibbs hydration energy of sulfate ions is larger than that of chloride ions [45]. The Hofmeister series and thermodynamic characteristics of sulfate and chloride ions suggest that the sorption of chloride ions on protamine molecules may be more preferential than that of sulfate ions. Sulfate ions are divalent and may induce crosslinking among protamine molecules and subsequent aggregation, whereas monovalent chloride ions may not. Similarly, sulfate ions change the solvation of protein complexes rather than chloride ions and subsequently induce aggregation [48].

The reason why protamine and plasmid DNA did not form complexes in PG (Figure S3) is a mystery, because the dielectric constant of PG is much lower than that of water (Table S4). The electrostatic interactions among the ions in PG would be stronger than those in water. This means that protamine and plasmid DNA could form complexes even in PG. The system contained anions (plasmid DNA, sulfate, and chloride ions) and cations (protamine and sodium ions). Plasmid DNA and protamine are macromolecules, and the viscosity of PG is higher than that of water. Considering the Brownian motion of plasmid DNA and protamine, the electrostatic interactions of small ions (sulfate, chloride, and sodium ions) with plasmid DNA and protamine may occur much faster than the interaction between plasmid DNA and protamine. PG, which has a high dipole moment, may prevent complexation through ion–dipole interactions. In particular, the formation of plasmid DNA/protamine complexes upon dilution of the alcoholic phase into the aqueous solution was confirmed (Figure S3). The interaction between the ions and water would also decrease the electrostatic interactions among the ions. The complex formation among plasmid DNA, protamine, and anionic lipids would increase the entropy in the system by releasing counterions, similar to DNA–protein complexes [49]. Although the theoretical basis of the NADIA method has not been fully proven, it would open the door to the large-scale production of ELNPs. Therefore, the most crucial achievement of this study was the discovery of the NADIA method.

**Scheme 1:**
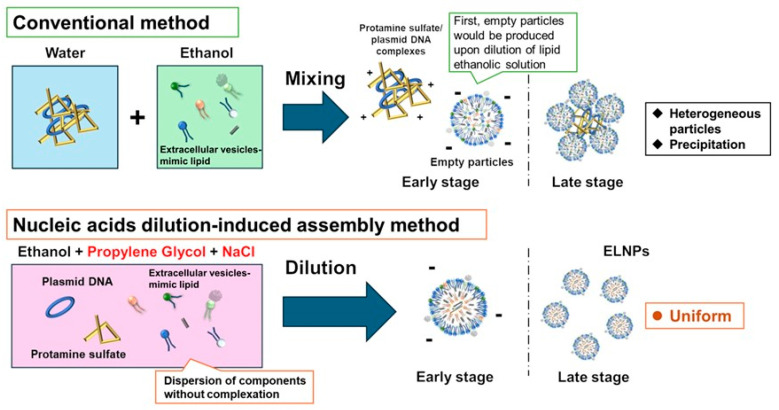
Schematic illustration of the conventional and nucleic acids dilution-induced assembly (NADIA) methods. In the conventional method, plasmid DNA is complexed with protamine in an aqueous solution. Upon dilution of the ethanol phase containing lipids, empty lipid nanoparticles (LNPs) are produced. The resulting empty LNPs form complexes with the protamine/plasmid DNA complexes. In the NADIA method, complexation of plasmid DNA, protamine, and lipids occurs upon dilution, enabling the formation of uniform ELNPs.

To achieve the stable formulation of homogeneous ELNPs, we hypothesized that it is necessary to determine the minimum N/P ratio because excess protamine sulfate would increase the levels of the free form of protamine sulfate, subsequently increasing the ζ-potential of ELNPs. Kanda et al. [50] reported the formation of complexes between nucleic acids and protamine sulfate at a N/P ratio of 1.6, demonstrating the high gene expression efficiency of γ-polyglutamic acids/protamine/plasmid DNA ternary complexes. We investigated the N/P ratio between protamine and plasmid DNA in detail and evaluated the minimum N/P ratio for sufficient complex formation based on an agarose gel retardation assay (Figure 2). The optimal N/P ratio of 1.8 was similar to that in the previous study [50]. In the conventional approach to form LNPs, protamine sulfate is mixed with plasmid DNA in an aqueous solution to form complexes and subsequently poured into a thin lipid film [51] or mixed with a lipid ethanol solution [52]. Such multiple processes are complex, making reproduction challenging. To address this, we developed the NADIA method, as mentioned above. In the NADIA method, the self-assembly of lipid nanoparticles occurs during dilution in the aqueous phase (Figure S3), reducing the number of preparation steps and enabling the formation of uniform nanoparticles (Scheme 1). To disperse plasmid DNA and protamine sulfate in the ethanol phase without precipitation, we explored methods to prevent their precipitation in alcohol (Figure 1, Figure 3 and Figure S3A). For this purpose, PG with 140 mM NaCl was the optimal choice. Gly was also able to disperse plasmid DNA and protamine sulfate; however, the physicochemical properties of the prepared ELNPs were not favorable (Figure S2). This may have been due to the high viscosity of Gly. Thus, we found that optimizing the N/P ratio, together with the selection of alcohols and the addition of the salt in the NADIA method, were all essential for the successful preparation of ELNPs.

Using the NADIA method, we successfully prepared uniform nanoparticles with small sizes (ca. 100 nm) that mimicked the lipid composition of EVs (Figure 4, Figure 5 and Figure 6). Recent advancements in microfluidic engineering have enabled the uniform and large-scale production of nanoparticles. Applying the developed EV-mimicking lipid nanoparticle preparation method to microfluidic engineering could facilitate GMP-standard mass production [53,54], contributing to the stable production and development of EV therapeutics. Applying the conditions revealed by the ethanol injection method, we explored parameters such as the FRR and TFR based on microfluidic engineering principles (Figure 6). Considering the slow injection speed of syringe pumps (Figure 5) and the study using microfluidic devices (Figure 6), an optimal TFR was suggested for the uniform production of core-shell nanoparticles. Excessive FRR and TFR led to the premature formation of LNPs before the core of protamine and plasmid DNA was established, resulting in a large PDI (Figure 4, Figure 5 and Figure 6). Conversely, insufficient ratios caused precipitation owing to electrostatic interactions, resulting in increased particle size and PDI. For particle size control, consideration of TFR and FRR together with alcohol and salt concentrations may be critical in future investigations about novel formulations of LNPs.

Regarding the structure of ELNPs, part of the plasmid DNA may localize on the surface of ELNPs, whereas most plasmid DNA may be in the inner core of ELNPs (Figure S7). It remains unclear how many plasmid DNA molecules are encapsulated per particle. Recently, Hara estimated nuclear size based on DNA density [55]. The ‘pseudo-nucleus’ calculated from the length of plasmid DNA using the reported equation was approximately 100–200 nm in diameter. This was almost equal to the size of ELNPs developed in this study, suggesting that one plasmid DNA molecule may be encapsulated per particle. Even though no free plasmid DNA was detected in the ELNPs (Figure S7, lane d), the encapsulation efficiency of plasmid DNA in the ELNPs was approximately 70% (Table S2). The remaining 30% were accessible to the external environment, indicating room for improvement.

Despite the absence of surface antigens and positive surface charge, ELNPs exhibited gene expression in HepG2 cells, comparable to that of cationic lipoplexes (Figure S8). Lipid raft-mediated endocytosis and macropinocytosis were involved in the mechanism of transfection using ELNPs (Figure 7). Similarly, native EVs are also taken up by cells via these mechanisms [56]. Our results suggest that the uptake of ELNPs is independent of surface antigens. The surface antigens of cell-derived EVs, such as tetraspanin, play crucial roles in cellular uptake and in vivo dynamics [57]. The uptake of EVs was increased even after the degradation of the membrane proteins in EVs by trypsin treatment [58], suggesting the possibility that factors other than surface antigens influence the uptake. Conventional EV preparation using cells makes it challenging to eliminate the influence of surface antigens. Applying our ELNPs could bypass this issue, allowing for the evaluation of EV function without interference from surface antigens. When HepG2 cells were treated with ELNPs, the transfection efficiency was comparable to that of cationic lipoplexes in the presence of serum. This implies that one or more lipids in EVs may contribute to cellular uptake via the interaction of ELNPs with serum proteins, forming a protein corona. Another possible reason for the uptake of ELNPs without surface antigens may be KRAS-dependent constitutive macropinocytosis [59]. HepG2 cells express the KRAS gene [60]. Therefore, macropinocytosis may be constitutively active in HepG2 cells, supporting the mechanism of transfection using ELNPs. Further investigation is required to elucidate these mechanisms.

## 5. Conclusions

We developed the NADIA method for preparing LNPs that mimicked the lipid composition of EVs. Through the simultaneous use of PG and NaCl for the alcohol phase, we discovered that the alcohol phase is suitable for dispersing nucleic acids, proteins, and lipids. This novel alcohol phase allows for the formulation of complex LNPs in a single alcohol injection into the aqueous phase. In the NADIA method, dilution of the alcohol phase with the aqueous phase triggered the self-assembly of the components. Combining the NADIA method with microfluidic devices decreased batch-to-batch variability in physicochemical and functional properties. Because microfluidic methods are reproducible and scalable, the NADIA methods discovered in this study would serve as a streamlined and reproducible platform for the high-quality and large-scale manufacture of complex formulations. Although ELNPs possessed negative ζ-potentials, their transfection efficiency was comparable to that of cationic lipoplexes. Our findings indicated that ELNPs led to gene expression through lipid raft-mediated endocytosis and micropinocytosis, despite the absence of surface antigens. This study underscores the importance of developing simple, robust, and reproducible preparation methods for complex formulations such as ELNPs, especially for potential therapeutic applications in gene delivery.

## Figures and Tables

**Table 1:** Physicochemical properties and transfection efficiencies of ELNPs prepared using the NADIA micropipette mixing method.

Preparation Number	Lipid/Plasmid DNA Ratio (µmol/10 µg)	Aqueous Phase/Alcohol Phase Ratio	Injection Speed (Q; 1.8, S; 0.18 mL/min)	Particle Size (nm)	SD	PDI	SD	ζ-Potential (mV)	SD	Gene Expression Efficiency (RLU/mL)	SD
M1	0.5	1	Q	124.9	3.05	0.27	0.036	−18.2	3.17	213,164,667	198,223,606
M2	0.5	1	S	144.4	9.15	0.25	0.058	−18.4	1.29	3,537,792	5,069,609
M3	0.5	2	Q	492.6	36.2	0.55	0.069	−21.7	1.39	81,792	10,815
M4	0.5	2	S	160.9	39.4	0.31	0.066	−19.2	0.96	72,042	4476
M5	0.5	3	Q	177.6	6.34	0.52	0.024	−19.5	1.44	275,458	312,114
M6	0.5	3	S	154.7	6.73	0.21	0.009	−16.5	0.91	82,917	11,064
M7	1.0	1	Q	161.4	6.19	0.36	0.029	−21.4	1.82	70,750	9443
M8	1.0	1	S	156.6	2.86	0.2	0.004	−19.6	1.28	217,958	251,479
M9	1.0	2	Q	230.7	26.1	0.43	0.065	−21.3	0.88	280,500	196,334
M10	1.0	2	S	240.8	20.1	0.54	0.045	−19.2	0.94	79,708	4195
M11	1.0	3	Q	213.8	17.5	0.48	0.124	−16	0.62	81,875	29,235
M12	1.0	3	S	161.6	5.90	0.31	0.046	−12	5.72	6,775,583	11,146,902
M13	1.5	1	Q	158.8	1.11	0.17	0.013	−19	0.52	2,251,458	3,510,584
M14	1.5	1	S	225.0	6.61	0.37	0.018	−17.7	1.01	704,708	1,028,941
M15	1.5	2	Q	141.1	3.13	0.25	0.036	−20.1	0.88	501,375	559,781
M16	1.5	2	S	175.7	2.40	0.28	0.029	−19.3	1.66	1,707,542	2,756,506
M17	1.5	3	Q	153.5	45.5	0.28	0.070	−20.5	16.2	2,672,792	4,389,694
M18	1.5	3	S	251.6	79.0	0.48	0.070	−16.4	0.51	97,042	20,721

Abbreviations: ELNPs, EV-mimicking lipid nanoparticles; NADIA, nucleic acids dilution-induced assembly; PDI, polydispersity index; RLU, relative light units.

**Table 2:** Physicochemical properties and gene expression efficiency of ELNPs prepared using the NADIA syringe pump method.

Preparation Number	Aqueous Phase/Alcohol Phase Ratio	Injection Speed (mL/h)	Particle Size (nm)	SD	PDI	SD	ζ-Potential (mV)	SD	Gene Expression Efficiency (RLU/mL)	SD
S1	1	1.0	135.2	1.00	0.22	0.005	−13.3	1.18	488,500	115,593
S2	2	1.0	172.1	1.65	0.43	0.016	−11.4	0.82	627,250	159,658
S3	3	1.0	165.1	4.44	0.44	0.021	−9.9	1.90	547,667	22,617
S4	1	6.5	143.4	0.09	0.31	0.032	−12.1	0.26	460,750	86,777
S5	2	6.5	144.3	5.32	0.28	0.039	−12.7	0.85	1,575,167	1,811,802
S6	3	6.5	150.2	1.90	0.34	0.024	−12.4	0.45	651,000	172,615
S7	1	12	148.5	4.22	0.31	0.024	−12.5	0.36	656,333	180,079
S8	2	12	142.0	2.59	0.23	0.036	−13.7	1.57	337,417	15,671
S9	3	12	142.4	2.71	0.22	0.026	−9.8	0.28	301,750	29,548

Abbreviations: ELNPs, EV-mimicking lipid nanoparticles; NADIA, nucleic acids dilution-induced induced assembly; PDI, polydispersity index; RLU, relative light units.

**Table 3:** Physicochemical properties and gene expression efficiency of ELNPs prepared using the NADIA microfluidic method.

Preparation Number	Aqueous Phase/Alcohol Phase Ratio	Injection Speed (mL/min)	Particle Size (nm)	SD	PDI	SD	ζ-Potential (mV)	SD	Gene Expression Efficiency (RLU/mL)	SD
MF1	1	1.0	157.0	2.07	0.27	0.029	−9.4	0.56	297,500	9014
MF2	2	1.0	102.2	1.04	0.14	0.014	−8.4	1.55	325,500	40,851
MF3	3	1.0	120.0	4.77	0.33	0.009	−8.9	0.89	296,083	17,680
MF4	1	6.5	86.8	0.33	0.17	0.008	−12.3	0.43	308,083	17,292
MF5	2	6.5	103.9	1.01	0.28	0.023	−10.4	1.18	325,667	25,891
MF6	3	6.5	102.1	2.44	0.31	0.040	−10.7	0.70	427,083	87,609
MF7	1	12	95.9	0.56	0.23	0.021	−12.3	0.57	620,250	254,971
MF8	2	12	94.4	2.76	0.23	0.026	−11.7	0.25	1,392,667	1,592,982
MF9	3	12	96.9	3.46	0.27	0.038	−10.6	0.96	414,750	40,002

Abbreviations: ELNPs, EV-mimicking lipid nanoparticles; NADIA, nucleic acids dilution-induced assembly; PDI, polydispersity index; RLU, relative light units.

## Data Availability

The data used in this study are available upon reasonable request.

## Supplementary Materials

The following supporting information can be downloaded at: https://www.mdpi.com/article/10.3390/cells13141183/s1, Figure S1: Dispersibility of protamine and plasmid DNA in water; Figure S2: Alcohol phases for protamine sulfate dispersion; Figure S3: Dispersion of protamine sulfate and plasmid DNA in alcohol phases and complex formation after dilution with the aqueous phase; Figure S4: Microscopic observation and turbidity measurement of the alcohol phase after lipid addition and subsequent dilution with the aqueous phase; Figure S5: Schematic diagrams for the preparation methods of ELNPs; Figure S6: Reproducibility of particle size and transfection efficiency of ELNPs prepared using micropipette and microfluidic NADIA methods; Figure S7: Agarose gel electrophoresis of ELNPs; Figure S8: Comparison of physicochemical properties and transfection efficiency of cationic lipoplexes and ELNPs; Table S1: Preparation conditions and physicochemical properties of ELNPs prepared using the NADIA micropipette mixing method with Gly; Table S2: Comparison of physicochemical properties and encapsulation efficiency of ELNPs prepared by the NADIA method with those prepared by other lipid nanoparticle and liposome preparation methods; Table S3: Dipole moments of solvents; Table S4: Dielectric constants of solvents [61,62].

## Abbreviations

1-Pro, 1-propanol; 2-Pro, 2-propanol; BSA, bovine serum albumin; Chol, cholesterol; CPZ, chlorpromazine hydrochloride; DIC, Differential interference contrast; DOPC, 1,2-dioleoyl-*sn*-glycero-3-phosphocholine; DOPE, 1,2-dioleoyl-*sn*-glycero-3-phosphoethanolamine; DOPS-Na, 1,2-dioleoyl-*sn*-glycero-3-phospho-L-serine, sodium salt; DOTAP, 1,2-dioleoyl-3-trimethylammonium propane; EIPA, ethyl-isopropyl-amiloride; EtBr, ethidium bromide; EtOH, ethanol; ELNPs, extracellular vesicles-mimicking lipid nanoparticles; EVs, extracellular vesicles; Gluc, *Gaussia princeps* luciferase; Gly, glycerol; LNPs, lipid nanoparticles; MβCD, methyl-β-cyclodextrin; NaCl, sodium chloride; NADIA, nucleic acids dilution-induced assembly; PEG, polyethylene glycol; PEG-Cer, N-octanoyl-sphingosine-1-{succinyl[methoxy(polyethylene glycol)2000]}; SM, sphingomyelin.
