# Supplementary material for: One-Step Formation Method of Plasmid DNA-Loaded, Extracellular Vesicles-Mimicking Lipid Nanoparticles Based on Nucleic Acids Dilution-Induced Assembly
Source: Cells. 2024 Jul 11;13(14):1183. doi: 10.3390/cells13141183 (PMC11274598; doi:10.3390/cells13141183)
Supplement: Supplementary file 1 [file cells-13-01183-s001.zip › cells-3058784-supplementary.pdf]

Supplementary Information

Cells

Article

One-Step Formation Method of Plasmid DNA-Loaded, Extracellular Vesicles-Mimicking Lipid Nanoparticles Based on Nucleic Acids Dilution-Induced Assembly

Kazuya Okami [1], Shintaro Fumoto [1,*], Mana Yamashita [1], Moe Nakashima [1], Hirotaka Miyamoto [1], Shigeru Kawakami [1] and Koyo Nishida [1]

[1] Graduate School of Biomedical Sciences, Nagasaki University, 1-7-1 Sakamoto, Nagasaki 852-8501, Japan
* Correspondence: sfumoto@nagasaki-u.ac.jp (S.F.)

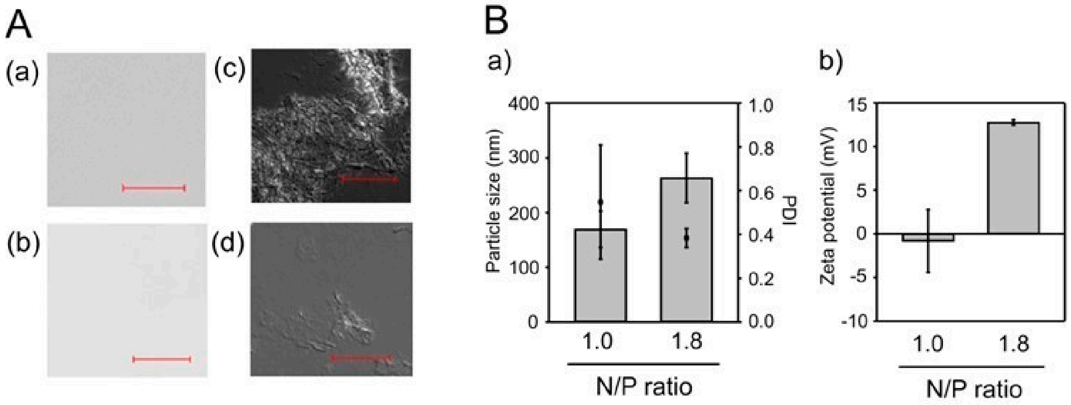

Figure S1. Dispersibility of protamine and plasmid DNA in water
A) Differential interference microscopy images of (a) plasmid DNA in water without NaCl, (b) protamine sulfate in water without NaCl, (c) protamine sulfate and plasmid DNA in water without NaCl, and (d) protamine sulfate and plasmid DNA in water containing 140 mM NaCl. B) (a) Particle size (bars), polydispersity index (PDI) (dots), and (b) zeta potential of protamine sulfate/plasmid DNA complexes prepared by mixing plasmid DNA with protamine sulfate in the presence of 140 mM NaCl. The N/P ratio is set at 1.0 and 1.8. Bars and dots represent the mean ± standard deviation of three experiments.

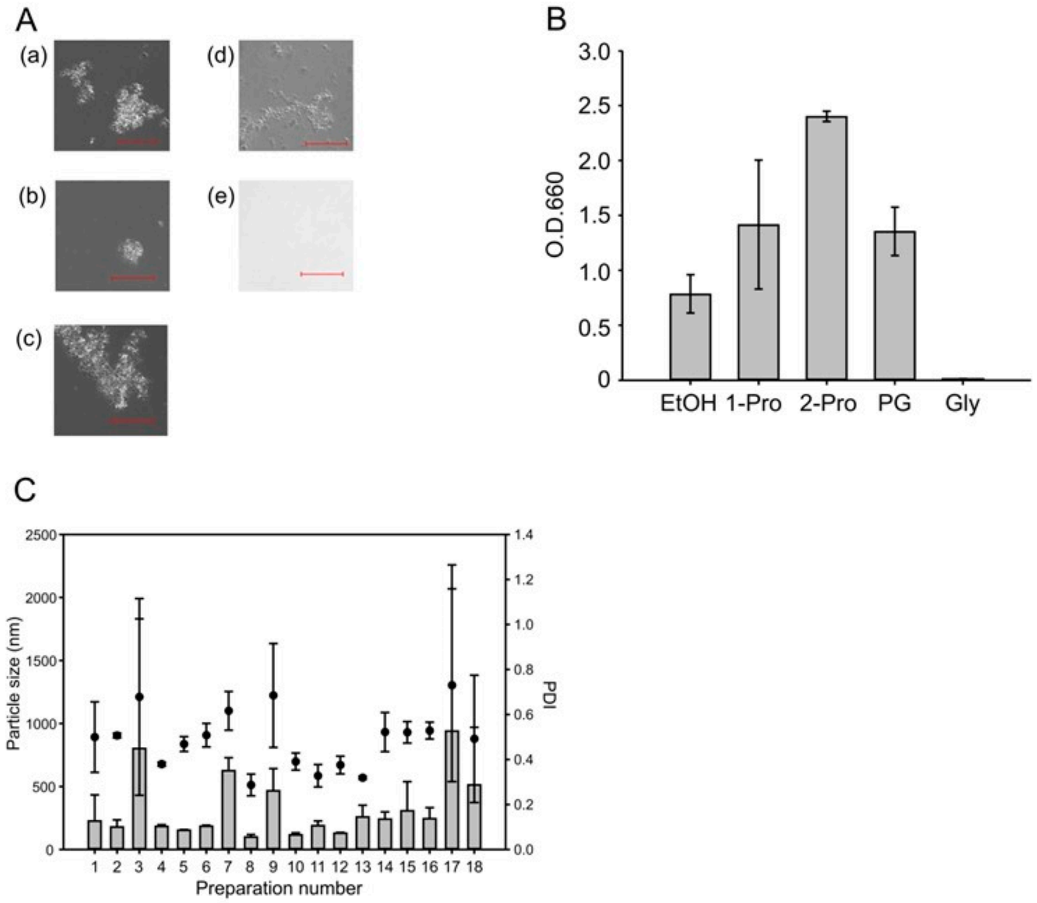

Figure S2. Alcohol phases for protamine sulfate dispersion

A) Differential interference microscopy images of protamine sulfate precipitation in various solvents. (a) 1-Propanol (1-Pro), (b) 2-Propanol (2-Pro), (c) Ethanol (EtOH), (d) Propylene glycol (PG), and (e) Glycerol (Gly). Scale bar, 50 μm. B) Turbidity measurement (optical density (O.D.) 660) of protamine sulfate in alcohols. C) Particle size (bars) and polydispersity index (PDI) (dots) of extracellular vesicle-mimicking lipid nanoparticles prepared using the nucleic acid dilution-induced assembly micropipette mixing method with Gly as the main component. Each number indicates the preparation conditions, including lipid amount (μmol), aqueous/alcohol ratio, and injection speed (Q, quick, ca. 1.8 mL/min; S, slow, ca. 0.18 mL/min). The preparation conditions are listed in Table S1.

Table S1. Preparation conditions and physicochemical properties of ELNPs prepared using the NADIA micropipette mixing method with Gly

Preparation number	Lipid/plasmid DNA ratio (μmol/10 μg)	Aqueous phase/alcohol phase ratio	Injection speed (Q; ca. 1.8, S; ca. 0.18 mL/min)	Particle size (nm)	PDI	ζ-potential (mV)
1	0.5	1	Q	229.0	0.50	−19.0
2	0.5	1	S	182.1	0.51	−19.6
3	0.5	2	Q	804.4	0.68	−15.4
4	0.5	2	S	185.8	0.38	−12.0
5	0.5	3	Q	156.2	0.47	−18.2
6	0.5	3	S	187.4	0.51	−17.6
7	1.0	1	Q	627.9	0.62	−21.2
8	1.0	1	S	102.7	0.29	−5.0
9	1.0	2	Q	468.8	0.68	−21.7
10	1.0	2	S	118.6	0.39	−19.8
11	1.0	3	Q	192.4	0.33	−12.9
12	1.0	3	S	132.3	0.38	−15.4
13	1.5	1	Q	260.9	0.32	−17.3
14	1.5	1	S	244.1	0.52	−18.2
15	1.5	2	Q	310.7	0.52	−17.6
16	1.5	2	S	247.6	0.53	−14.7
17	1.5	3	Q	942.4	0.73	−14.7
18	1.5	3	S	516.1	0.49	−13.5

Abbreviations: ELNPs, extracellular vesicles-mimicking lipid nanoparticles; NADIA, nucleic acid dilution-induced assembly; Gly, glycerol; PDI, polydispersity index.

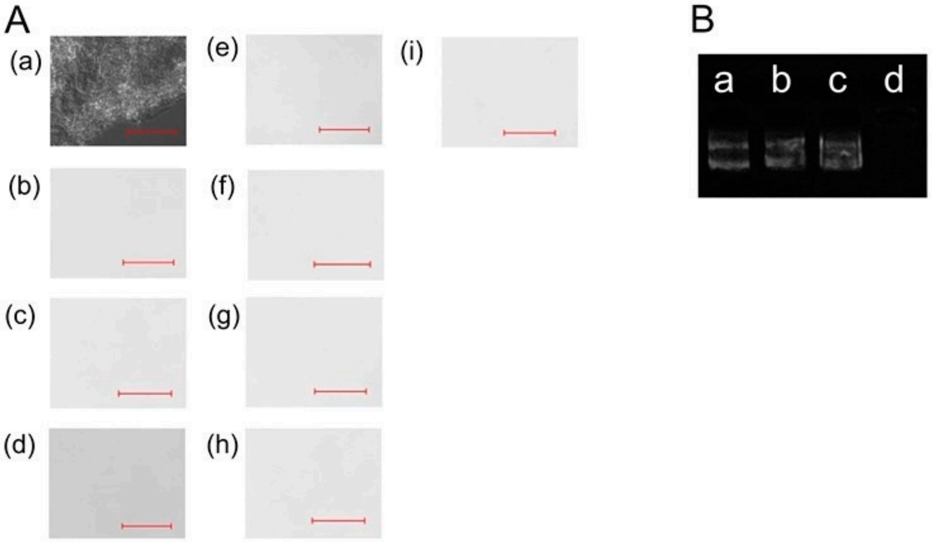

Figure S3. Dispersion of protamine sulfate and plasmid DNA in alcohol phases and complex formation after dilution with aqueous phase

A) Differential interference microscopy images of the precipitation of protamine sulfate and plasmid DNA at various ethanol (EtOH) and propylene glycol (PG) ratios. (a) EtOH/PG= 9/1, (b) EtOH/PG= 8/2, (c) EtOH/PG= 7/3, (d) EtOH/PG= 6/4, (e) EtOH/PG= 5/5, (f) EtOH/PG= 4/6, (g) EtOH/PG= 3/7, (h) EtOH/PG= 2/8, and (i) EtOH/PG= 1/9 with NaCl (140 mM). Scale bar, 50 μm. B) Formation of protamine sulfate/plasmid DNA complexes after dilution with aqueous phase. An agarose gel retardation assay is performed. Plasmid DNA is dispersed in EtOH/PG (1/1, v/v) with 140 mM NaCl (a) and then mixed with acetate buffer (100 mM, pH 4.4) (b). Plasmid DNA and protamine sulfate are dispersed in EtOH/PG (1/1, v/v) with 140 mM NaCl (c) and then mixed with acetate buffer (100 mM, pH 4.4) (d). Each well contains 500 ng plasmid DNA.

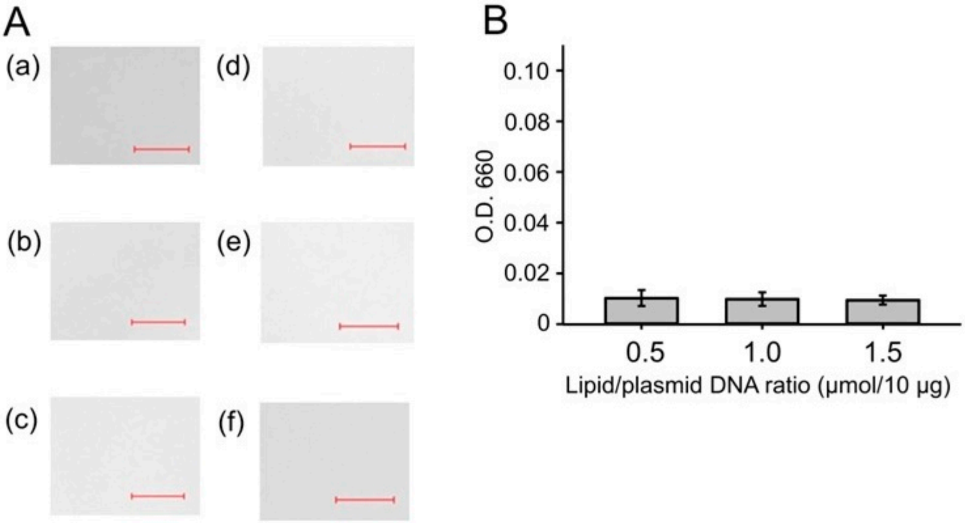

Figure S4. Microscopic observation and turbidity measurement of the alcohol phase after lipid addition and subsequent dilution with aqueous phase

A) Differential interference microscopy images of the alcohol phases (ethanol (EtOH)/propylene glycol (PG),1/1 with NaCl 140 mM, 300 μL) after adding lipids (a, b, c), and dilution with the aqueous phase (acetate buffer 100 mM, pH 4.4, 300 μL) (d, e, f). Lipid/plasmid DNA ratios (μmol/10 μg) are 0.5 (a, d), 1.0 (b, e), and 1.5 (c, f). B) Turbidity measurement of alcohol phases (EtOH/PG, 1/1 with NaCl 140 mM) at various lipid/plasmid DNA ratios (μmol/10 μg). Each bar represents the mean ± standard deviation of three experiments. O.D., optical density

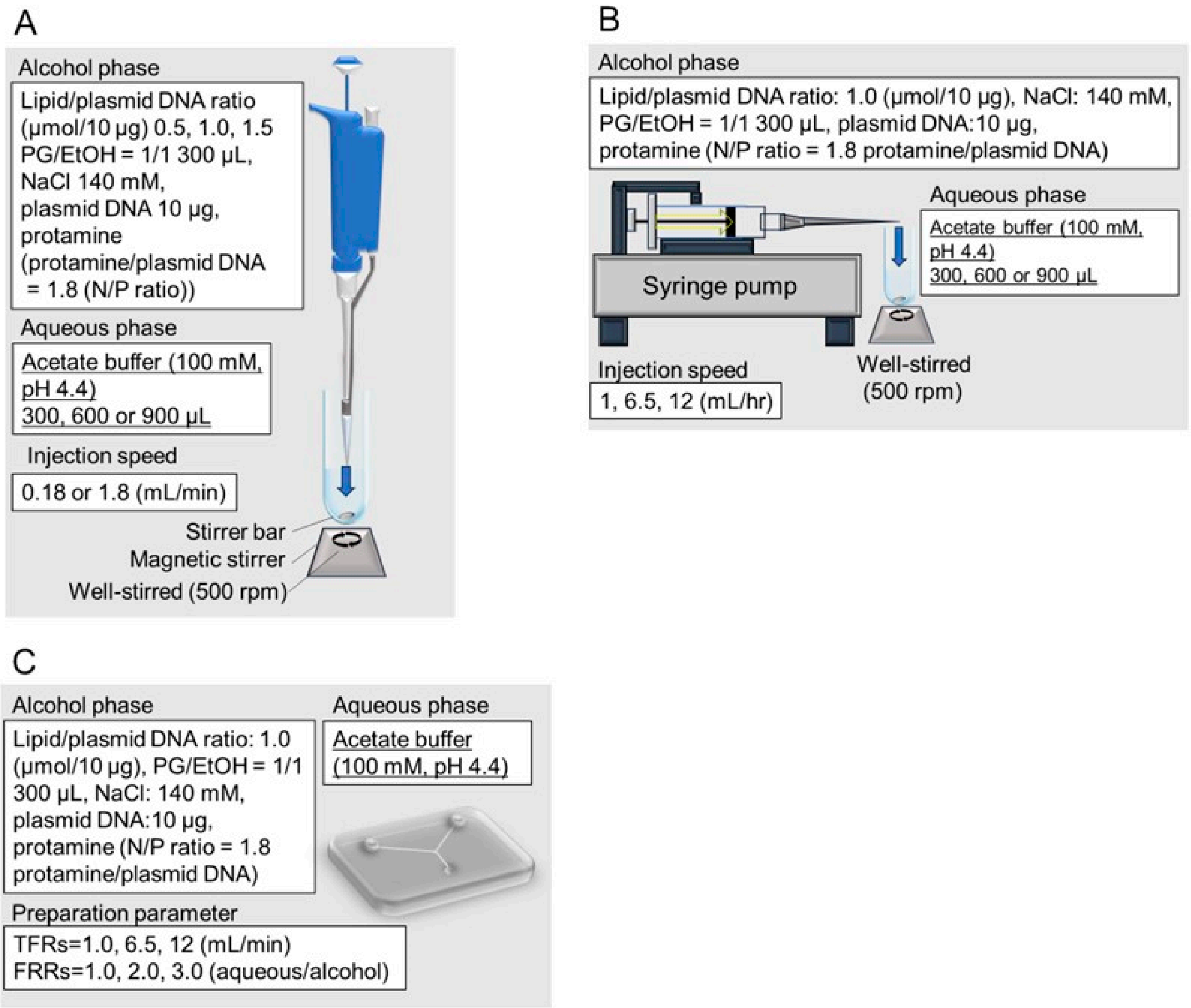

Figure S5. Schematic diagrams for the preparation methods of ELNPs

A) Micropipette mixing, B) syringe pump, and C) microfluidic procedure based on the NADIA method. ELNPs, extracellular vesicle-mimicking lipid nanoparticles; NADIA, nucleic acid dilution-induced assembly; PG, propylene glycol; EtOH, ethanol; TFR, total flow rate; FRR, flow rate ratio

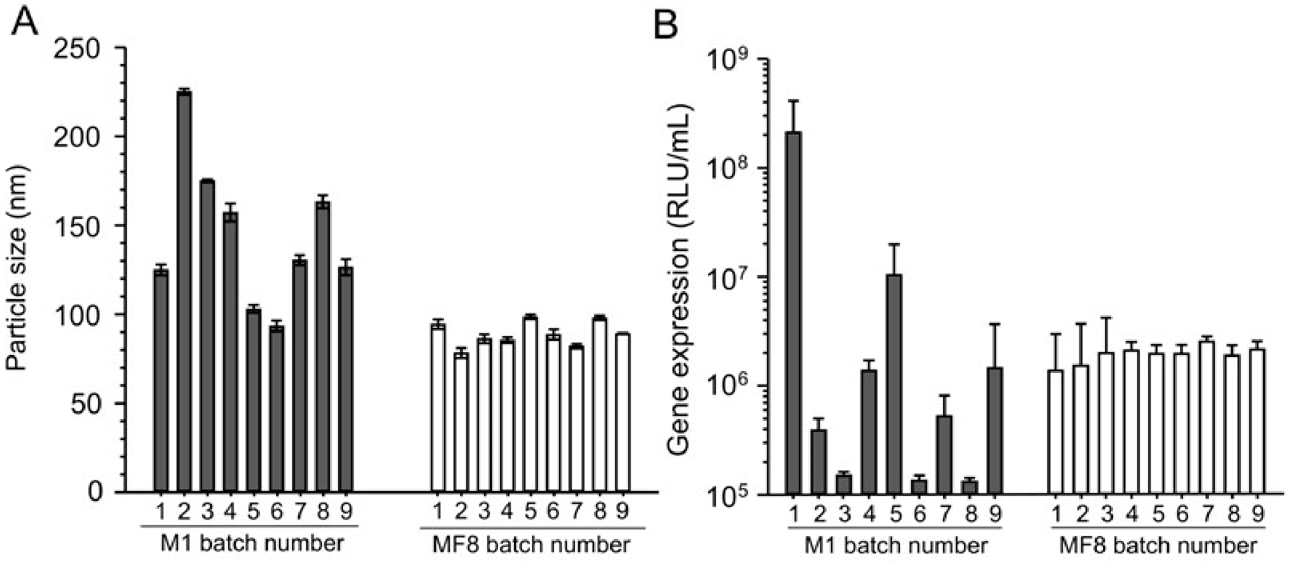

Figure S6. Reproducibility of particle size and transfection efficiency of ELNPs prepared using micropipette and microfluidic NADIA methods

A) Particle size. Each bar and dot represent the mean ± S.D. of three replicates. B) Transfection efficiency. Each bar represents the mean + S.D. of three replicates. Extracellular vesicles-mimicking lipid nanoparticles (ELNPs) were prepared nine times and designated as Batch1 - 9. NADIA, nucleic acids dilution-induced assembly; RLU, relative light unit.

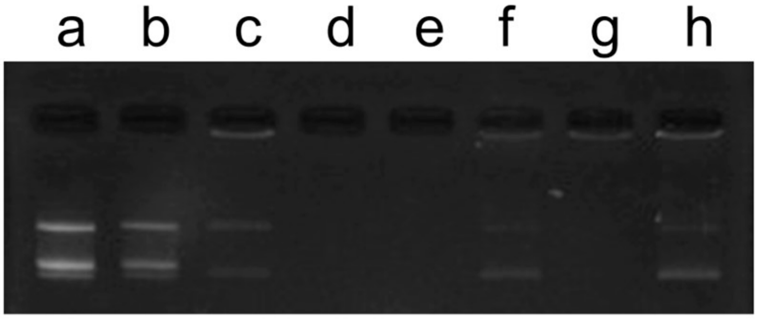

Figure S7. Agarose gel electrophoresis of ELNPs

a) Naked plasmid DNA 100 ng, b) naked plasmid DNA 50 ng, c) ELNPs with plasmid DNA 50 ng without protamine sulfate, d) ELNPs with plasmid DNA 50 ng and protamine sulfate, e) ELNPs with plasmid DNA 50 ng and protamine sulfate after incubation with PBS(-), f) ELNPs with plasmid DNA 50 ng and protamine sulfate after incubation with PBS(-) containing 0.25% (w/w) Triton X-100, g) ELNPs with plasmid DNA 50 ng and protamine sulfate after incubation with PBS(-) containing 0.008% (w/v) dextran sodium sulfate, h) ELNPs with plasmid DNA 50 ng and protamine sulfate after incubation with PBS(-) containing 0.25% (w/w) Triton X-100 and 0.008% (w/v) dextran sodium sulfate. The selected ELNPs were MF8 in the main text. Abbreviations: ELNPs, extracellular vesicles-mimicking lipid nanoparticles.

Table S2. Comparison of physicochemical properties and encapsulation efficiency of ELNPs prepared by the NADIA method with those prepared by other lipid nanoparticle and liposome preparation methods

	Particle size (nm)	S.D.	PDI	S.D.	ζ-potential (mV)	S.D.	Encapsulation efficiency (%)	S.D.
Bangham method								
Empty	369.8	9.29	0.70	0.11	−10.53	0.56	N.A.	
Protamine/plasmid DNA	457.7	62.5	0.52	0.08	−6.39	0.56	54.95	5.56
Conventional ethanol injection								
Empty	206.5	1.39	0.05	0.01	−7.14	0.73	N.A.	
Protamine/plasmid DNA	208.0	3.27	0.29	0.03	−0.74	1.37	21.59	11.60
NADIA micropipette mixing								
M1 empty	106.3	2.12	0.22	0.01	−10.80	1.66	N.A.	
M1 Protamine/plasmid DNA	140.3	2.82	0.23	0.01	−5.85	1.35	61.19	8.91
NADIA microfluidic mixing								
MF8 empty	126.4	4.37	0.57	0.11	−12.34	1.11	N.A.	
MF8 Protamine/plasmid DNA	82.1	1.30	0.24	0.02	−7.86	2.56	71.49	5.55

N.A.: not applicable. Abbreviations: ELNPs, extracellular vesicles-mimicking lipid nanoparticles; NADIA, nucleic acid dilution-induced assembly; PDI, polydispersity index

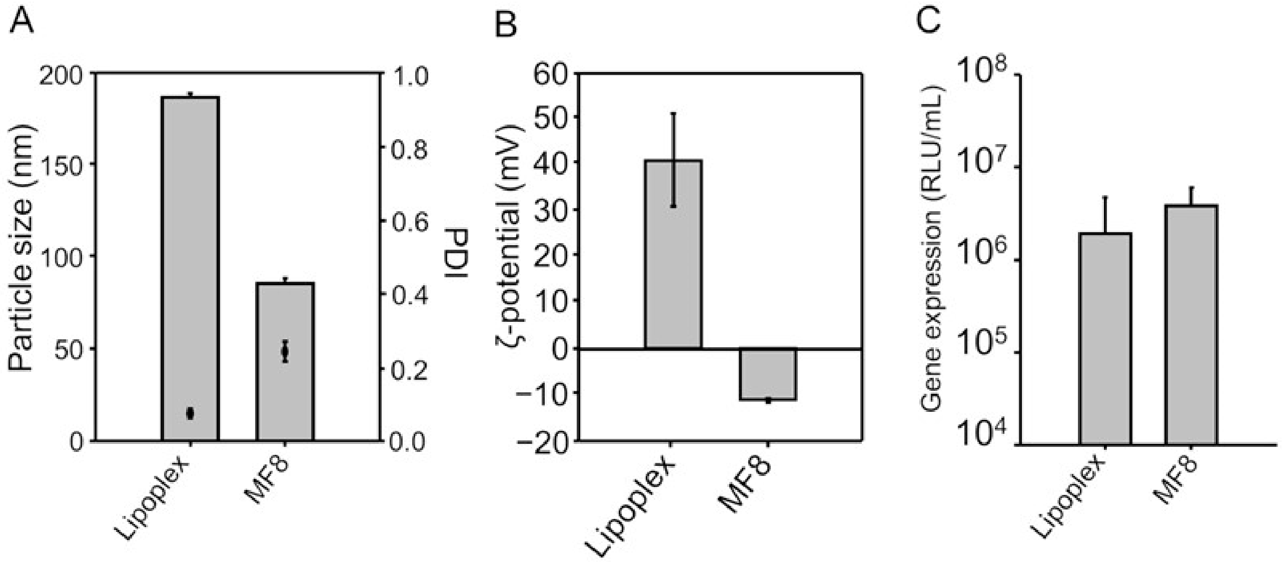

Figure S8. Comparison of physicochemical properties and transfection efficiency of cationic lipoplexes and ELNPs
The cationic lipoplexes (1,2-dioleoyl-3-trimethylammonium propane / cholesterol 1:1) are prepared by mixing plasmid DNA into cationic liposomes. The charge ratio is set as 2.3. The exosome-mimicking lipid nanoparticles (ELNPs) are prepared using the nucleic acid dilution-induced assembly microfluidic method (MF8, as shown in Table 3). A) Particle size (bars) and polydispersity index (PDI) (dots). B) ζ-potential. Bars and dots represent the mean ± standard deviation (S.D.) of three experiments. C) Transfection efficiency in HepG2 cells. Each bar represents the mean + S.D. of three experiments. RLU, relative light unit

Table S3. Dipole moments of solvents

Solvent	Dipole moment (Debye)
Water	2.308
Ethanol	1.773
1-Propanol	1.732
2-Propanol	1.726
Propylene glycol	2.793
Glycerol	2.906

The dipole moments of the solvents are calculated based on the second-order Møller–Plesset perturbation theory (calculation at the 3-21G level) using the online calculation site (https://cccbdb.nist.gov/).

Table S4. Dielectric constants of solvents

Solvent	Dielectric constant	Reference
Water	78.5	[61]
Ethanol	24.3	[61]
1-Propanol	20.8	[62]
2-Propanol	18.6	[62]
Propylene glycol	32.0	[61]
Glycerol	40.1	[61]
